# Expert Consensus on the Use of Diphenhydramine for Short-Term Insomnia: Efficacy, Safety, and Clinical Applications

**DOI:** 10.3390/jcm14103297

**Published:** 2025-05-09

**Authors:** Daniel Felipe Ariza-Salamanca, Marco Venegas, Karem Parejo, Steve Amado, Jorge Echeverry, Carlos Alberto Calderón-Ospina

**Affiliations:** 1 Department of Pharmacobiology, Center for Research and Advanced Studies (Cinvestav), National Polytechnic Institute, Mexico City 14300, Mexico; daniel.ariza@cinvestav.mx; 2 Clinic for the Study and Treatment of Sleep Disorders Somnarum, Sleep and Electrodiagnostics Unit, Bogota 111031, Colombia; marcoaureliovenegas@gmail.com; 3 Sleep Laboratory, Fundación Clínica Shaio, Bogota 110131, Colombia; karemparejo@gmail.com; 4 Maple Respiratory Colombia, Sleep Clinic, Bogota 111211, Colombia; steveamadog@gmail.com (S.A.); jechever@interco.net.co (J.E.); 5 Faculty of Health Sciences, Technological University of Pereira, Pereira 660003, Colombia; 6 Center for Research in Genetics and Genomics (CIGGUR), Institute of Translational Medicine (IMT), School of Medicine and Health Sciences, Universidad del Rosario, Bogotá 111221, Colombia; 7 Research Group in Applied Biomedical Sciences (UR Biomed), School of Medicine and Health Sciences, Universidad del Rosario, Bogotá 111221, Colombia

**Keywords:** diphenhydramine, antihistamines, short-term insomnia, experts’ consensus

## Abstract

Insomnia is the most prevalent sleep disorder, estimated to affect at least one-third of the global population. There are a variety of treatment options available for both acute and chronic insomnia. Currently, the pharmacological arsenal for treating insomnia includes short- or intermediate-acting benzodiazepine hypnotics, non-benzodiazepine hypnotic sedatives, melatonin receptor agonists, orexin receptor antagonist, and sedating antidepressants. Diphenhydramine, a first-generation antihistamine, is commonly used in the treatment of allergies and dermatitis. This review examines the preclinical and clinical efficacy and safety evidence of diphenhydramine in treating short-term insomnia. Additionally, it provides expert consensus on its implementation as an over-the-counter medication for this condition. The available evidence indicates that diphenhydramine is an effective treatment for acute insomnia in adults, offering a safe and affordable option for most patients suffering from this condition. Experts concur that there is strong evidence supporting the recommendation of diphenhydramine for the treatment of acute insomnia in adults.

## 1. Introduction

Insomnia is characterized by difficulties in initiating or maintaining sleep, early morning awakenings, and dissatisfaction with the quality or quantity of sleep despite adequate opportunity to sleep. It is commonly associated with daytime impairments such as fatigue, mood disturbances, or cognitive difficulties [1]. Insomnia is a prevalent sleep disorder worldwide [2]. For instance, in the United States, a survey of 7428 adults revealed that nearly half reported difficulty sleeping, with an estimated prevalence of insomnia at 23.2% [3]. In Latin America, a study conducted in four major cities—Montevideo, Mexico City, Santiago, and Caracas—examined 4533 participants and found a high prevalence of symptoms related to sleep disorders, including 34.7% diagnosed with insomnia [4]. In Colombia, a study of 1325 women from diverse ethnic backgrounds reported that nearly one-third experienced insomnia [5].

It is recognized as a disease on its own since it significantly impairs quality of life, daytime functioning, and overall health. Therefore, it should be promptly treated once detected. Cognitive behavioral therapy for insomnia (CBT-I) is widely recognized as the first-line therapy [6]. Pharmacotherapy is also a helpful tool for treating insomnia; it has demonstrated to improve significantly latency to sleep and awakenings [7]. The choice of medication is a critical aspect of insomnia management, as tailoring treatment to the individual can optimize outcomes and minimize risks. While widely approved medications for insomnia, such as benzodiazepines and Z-drugs, are commonly prescribed, other effective options, like antihistamines, are not as broadly endorsed despite their potential benefits.

This article provides a comprehensive review of insomnia, focusing on the available literature regarding diphenhydramine for the short-term management of insomnia. It includes an expert consensus from various medical specialties on the use of diphenhydramine, with particular emphasis on its safety, efficacy, and special considerations in children and the elderly. Finally, it contrasts these findings with the existing literature, offering a nuanced perspective on the role of diphenhydramine in the treatment of short-term insomnia.

### 1.1. Pathophysiological and Clinical Aspects of Insomnia

There is no definitive consensus on the biological basis of insomnia. Nonetheless, hyperarousal is widely accepted as a primary biological mechanism. From a biological perspective, hyperarousal results from the overactivation of the ascending reticular activation systems and the hypothalamic–pituitary–adrenal (HPA) axis [8,9]. It manifests as an elevated heart rate, abnormal heart rate variability, altered core body temperature, and blunted reductions in the metabolic rate typically associated with non-rapid eye movement (non-REM) sleep [10].

The Spielman model posits that insomnia is influenced by three factors: predisposing factors (genetic, personality, or environmental traits that increase vulnerability), precipitating factors (acute events, such as trauma), and perpetuating factors (behavioral and cognitive patterns that sustain insomnia and lead to chronic forms) [11].

Insomnia is diagnosed clinically based on various criteria, including those outlined in the International Classification of Sleep Disorders, Third Edition (ICSD-3); the Diagnostic and Statistical Manual of Mental Disorders, Fifth Edition (DSM-5); and the International Classification of Diseases, Tenth Revision (ICD-11). According to the ICSD-3, insomnia is defined as a persistent difficulty in initiating or maintaining sleep, or waking earlier than desired, accompanied by resistance to going to bed at an appropriate time or inability to sleep without external intervention, despite having adequate opportunities and conditions for sleep [12].

The diagnosis of insomnia can be achieved through a comprehensive clinical evaluation that includes a detailed sleep history, focusing on the onset, duration, and frequency of symptoms. Current sleep–wake patterns, along with environmental and social factors, should be assessed. A thorough medical and psychiatric history is essential, as well as an evaluation of substance use and medication intake [10].

In addition to clinical diagnosis, there are supportive diagnostic tools that, while not strictly necessary, can provide valuable information. Sleep diaries are recommended, as they offer insights into sleep efficiency and patterns [13]. Polysomnography (PSG) is particularly useful when other sleep disorders are suspected, as it can reveal findings characteristic of insomnia, such as disrupted sleep continuity [14]. Actigraphy can also be helpful, as it provides objective data on sleep state misperception and paradoxical insomnia [15,16]. Furthermore, modern smart technologies, such as smartwatches, are increasingly used to monitor sleep patterns and can serve as practical tools to support the follow up of patients diagnosed with insomnia [17].

### 1.2. Treatment of Insomnia

Treatment includes both pharmacological and behavioral interventions [18]. Behavioral interventions emphasize strict sleep hygiene and psychological approaches, such as CBT-i and sleep restriction therapy [12]. Although CBT-i has been demonstrated to improve sleep, it is not widely available in all regions. Therefore, it is crucial for the primary care physician to understand what to teach patients presenting with insomnia. Sleep hygiene, body–mind therapy, meditations, mindfulness, and diaphragmatic breathing are also available interventions for managing insomnia [6]. Primary care physicians should also be confident in the use of medications to manage insomnia.

Pharmacological options for treating insomnia include short- or intermediate-acting benzodiazepine (BDZ) hypnotics, non-benzodiazepine hypnotic sedatives (Z drugs), melatonin receptor agonists, orexin antagonists, and sedating antidepressants. The pharmacodynamics of these drugs can be categorized into GABAergic and non-GABAergic types. Most of them are detectable in plasma within 30 min of ingestion and have short to medium half-lives. As these drugs are hypnotic, they interact with other substances such as alcohol. Special populations, such as children and the elderly, must be considered when selecting a drug. Table 1 summarizes the main pharmacological features of currently used drugs for treating insomnia.

The use of diphenhydramine for short-term insomnia is a key focus of this consensus. The approval of diphenhydramine as an over-the-counter medication by the Food and Drug Administration, along with strong evidence supporting its effectiveness for short-term insomnia and the safety profile offered, has driven this research. Subsequent sections explore the available literature on the general pharmacological properties of diphenhydramine. To assess the accuracy and quality of the evidence, a consensus committee was assembled. Additionally, considerations are provided regarding its implementation as an over-the-counter treatment for short-term insomnia in Colombia.

## 2. Materials and Methods

### 2.1. Selection of Consensus Committee Members and Topics Being Assessed

A panel of five experts in sleep disorders was convened to participate in this consensus. CAC-O is a pharmacologist specializing in pharmacovigilance, with extensive experience in researching neurological disorders. MV and KJPG are neurologists specialized in sleep disorders, YSA is an otolaryngologist specializing in sleep disorders, and JEEC is a psychiatrist specializing in sleep disorders. Most of these experts are clinicians who regularly treat patients with various sleep disorders and have a deep understanding of central nervous system disorders, as well as high academic qualifications.

The objectives of this consensus were threefold: to gather the opinions of Latin American specialists on key issues related to the use of diphenhydramine in short-term insomnia, to thoroughly evaluate the available literature on the drug’s efficacy and safety, and to delineate the clinical scenarios in which diphenhydramine is or is not an optimal choice.

Each expert was assigned to review the selected literature, chosen through a quasi-systematic search. The reviewed studies were then carefully discussed, and a questionnaire was developed based on the most recent insomnia taxonomy. The key topics, selected in consultation with the committee, included drug effectiveness and safety, availability, patient age, treatment duration, and the level of evidence supporting each recommendation.

### 2.2. Literature Research

The literature search was carried out by scanning Medline for the Medical Subject Heading (MeSH) “Insomnia” AND “Diphenhydramine” via PubMed, which showed 115 results. There were no limitations in terms of the type and quality of studies, language, or provenance. The title and abstract of each article were screened for their relevance to the current approach by CA-O. The articles directly related to diphenhydramine and acute insomnia that were considered relevant were selected and reviewed. The date of the last PubMed literature search was 30 May 2024. Regarding the exclusion of 79 articles, which reduced the selection from 115 to 36, this was primarily since many of the initially retrieved articles were not directly related to the use of diphenhydramine for the management of insomnia. Instead, they focused on antihistamines in general or addressed indications for diphenhydramine unrelated to insomnia. A total of 36 articles were chosen to provide the evidence support for this consensus.

### 2.3. Consensus Workflow and Methods to Achieve Consensus

The Delphi methodology was followed to reach a consensus among a panel of experts on the topic [19]. This process included the selection of a group of qualified experts, who participated in several rounds of questionnaires designed to collect their opinions and judgments. After each round, anonymous and summarized feedback was provided to the group, allowing the experts to review and adjust their responses based on the input from the collective. This iterative process continued until a consensus was reached in most of the questions. Finally, the results were analyzed to obtain consensual conclusions that reflected the general agreement of the panel.

Experts were assigned at least five articles each to review in preparation for an upcoming virtual meeting. During the meeting, different aspects of diphenhydramine were thoroughly assessed by all experts, considering the selected literature. A draft of the questionnaire was then reviewed and refined for proper taxonomy and syntax. A total of 12 questions were formulated (Box 1).

Box 1 **Questionnaire regarding the use of diphenhydramine in short-term insomnia.**

Chapter 1: Evaluation of the use of diphenhydramine for insomnia: efficacy, safety, convenience, and cost of diphenhydramine in the management of insomnia:
   1. Diphenhydramine is an effective medication for the management of short-term insomnia.
   2. Diphenhydramine is a safe medication for the management of short-term insomnia.
   3. If diphenhydramine were available in the Colombian market, do you consider that this medication could be an accessible option for managing short-term insomnia?
   4. Diphenhydramine is a convenient medication for most patients with acute insomnia, regardless of their comorbidities or clinical situations, and therefore, has the potential to be marketed as an over-the-counter medication for managing short-term insomnia.
Chapter 2: type(s) of insomnia where diphenhydramine could be used:
   5. Diphenhydramine is a useful medication for short-term insomnia (less than 3 months in duration).
   6. Diphenhydramine is a useful medication for chronic insomnia (more than 3 months in duration).
Chapter 3: use of diphenhydramine as a hypnotic/sedative by age group:
   7. Diphenhydramine is an effective and safe medication for children and adolescents (7 to 17 years old) for managing short-term insomnia.
   8. Diphenhydramine is an effective and safe medication for young adults (18 to 65 years old) for managing short-term insomnia.
   9. Diphenhydramine is an effective and safe medication for elderly individuals (65 years and older) for managing short-term insomnia.
Chapter 4: duration of diphenhydramine treatment for managing insomnia:
   10. The maximum recommended duration for using diphenhydramine as a hypnotic/sedative for short-term insomnia should be around four weeks.
Chapter 5: evidence and levels of evidence on the use of diphenhydramine for managing short-term insomnia:
   11. There is a sufficient body of clinical evidence to recommend the use of diphenhydramine in patients with short-term insomnia.
   12. There is a sufficient level of clinical evidence to recommend the use of diphenhydramine in patients with short-term insomnia.

The questionnaire was divided into five chapters. Chapter 1 focused on the evaluation of the use of diphenhydramine for insomnia, including its efficacy, safety, convenience, and cost in the management of insomnia. Chapter 2 addressed the types of insomnia in which diphenhydramine could be used. Chapter 3 examined the use of diphenhydramine as a hypnotic/sedative by age group. Chapter 4 considered the duration of diphenhydramine treatment for managing insomnia. Chapter 5 explored the evidence and levels of evidence on the use of diphenhydramine for managing short-term insomnia.

All questions were quantified by a Likert Scale, with 1= total disagreement, 2 = partial disagreement, 3 = neutral, 4 = partial agreement, and 5 = total agreement. An agreement of less than 60% of the votes was considered as no agreement, a supermajority between 60% and 74% was considered a weak agreement, a supermajority equal or greater than 75% as a strong agreement, and 100% as unanimous agreement. A consensus was defined as an interquartile range equal to or less than 1. The interquartile range (IQR) was calculated by finding the difference between the third quartile (Q3) and the first quartile (Q1) (IQR = Q3 − Q1). All the answers were treated anonymously. Statistical analyses and figures were performed using MATL–AB Online. Version R2022b (MathWorks, Natick, MA, USA).

## 3. Results and Discussion

In this section, we present the results of the literature review on various aspects of diphenhydramine use for short-term insomnia. Additionally, we provide an analysis of the frequency and agreement among experts regarding each question within the same dimensions explored in the literature.

### 3.1. Diphenhydramine Pharmacodynamics and Efficacy in Insomnia

Diphenhydramine is a first-generation antihistamine that was discovered in the 1940s [20]. Since its introduction to the market, it has been widely used for the treatment of various allergic conditions, including allergic rhinitis, urticaria, and dermatitis [21]. Diphenhydramine antagonizes the H1 histamine receptor. Histamine receptors have distinct roles and locations: H1 and H2 receptors are postsynaptic and excitatory, with H1 linked to phospholipase C and found in the hypothalamus and limbic regions, while H2 is coupled to adenylate cyclase and concentrated in the hippocampus, amygdala, and basal ganglia. In contrast, H3 receptors are presynaptic, inhibitory, and primarily located in the basal ganglia, regulating histamine and neurotransmitter release by inhibiting calcium channels [22].

H1 receptors are G-protein-coupled receptors (GPCRs) linked to the Gq pathway, which activates phospholipase C, leading to the inositol triphosphate (IP3) and diacylglycerol (DAG) signaling cascade, ultimately enhancing the neural activity. These receptors exhibit high basal activity and induce cortical desynchronization, a state associated with heightened brain activity and wakefulness [23,24]. Diphenhydramine acts as a negative allosteric modulator and inverse agonist of H1 receptors, blocking histamine’s action at these sites. By inhibiting histamine binding, it reduces neuronal excitation mediated by H1 receptors, decreasing the cortical activity and inducing sleepiness [25].

Diphenhydramine can easily cross the blood–brain barrier due to its lipophilic nature. Once in the brain, its sedative effect is enhanced by its ability to interact with other neurotransmitter systems other than the histaminergic. Although its affinity is lower than H1, diphenhydramine can also affect muscarinic acetylcholine receptors, which also play a role in regulating the sleep–wake cycle. The blockade of these receptors contributes to the secondary sedative effect [26].

For instance, Carruthers et al. demonstrated in healthy volunteers that a 50 mg dose of diphenhydramine exhibited a positive correlation between the plasma concentration and sedative effects [27]. Similarly, Roth et al. compared the effects of diphenhydramine (50 mg TID) and loratadine (10 mg and 40 mg) in 16 healthy adults. Diphenhydramine significantly reduced sleep latency, but was associated with an impaired daytime performance. However, it is worth noting that diphenhydramine was used at high doses in this study and the patients took the medication every 8 h (including two daytime doses) instead of taking the medication at night, before going to sleep [28]. Moreover, Boberly et al. recruited healthy young adult volunteers who received 50 to 75 mg of diphenhydramine. Self-reported sleep latency showed mild hypnotic effects, with no significant differences in subjective sleep parameters, and no deterioration in the psychomotor performance or rebound insomnia [25]. Similar findings have been reported in healthy individuals [29,30].

Going deeper into diphenhydramine evidence for insomnia, Rickels et al. conducted a double-blind, crossover study to evaluate the effect of diphenhydramine on insomnia in adults. They compared 50 mg of diphenhydramine with a placebo in 111 patients with mild to moderate insomnia. Significant improvements were observed in sleep latency and restfulness with diphenhydramine. Furthermore, the authors recommended diphenhydramine as an over-the-counter sleep aid in the treatment of temporary mild to moderate insomnia [31]. Accordingly, Morin et al. reported improvements in subjective sleep parameters and increased sleep efficiency in 184 patients with mild insomnia who received 50 mg of diphenhydramine twice daily [32]. Schweitzer et al. compared drowsiness and performance levels between two antihistamines, diphenhydramine, and cetirizine. This study administered 50 mg of diphenhydramine, 10 mg/day of cetirizine, or a placebo three times daily for three days. Twelve atopic subjects received each treatment in a double-blind Latin square design. The main findings indicated that diphenhydramine, unlike cetirizine, caused acute decreases in alertness and performance. However, tolerance to its sedative effects developed by day three, suggesting that diphenhydramine may be useful for short-term insomnia [33].

Regarding studies evaluating the efficacy of diphenhydramine in different populations, in the elderly, Teutsch et al. compared the hypnotic effects of diphenhydramine and methapyrilene with those of pentobarbital in hospitalized veterans. The main findings indicated that 50 mg or 150 mg of diphenhydramine were no more effective than 60 mg of pentobarbital in treating insomnia, meaning diphenhydramine was no different to pentobarbital to induce sleep [34]. Similar findings were obtained by Glass et al. [35]. Furthermore, Stewart et al. conducted a randomized, double-blind, crossover clinical trial to test 50 mg of diphenhydramine and 15 mg of temazepam. The main results showed that diphenhydramine reduced sleep latency more effectively than the placebo, provided a longer sleep duration than temazepam on the fifth night, and both temazepam and diphenhydramine were associated with residual daytime drowsiness [36].

In the case of diphenhydramine use in children with sleep disorders, Russo et al. conducted a double-blind, placebo-controlled trial of diphenhydramine at 10 mg/kg. The main findings showed that diphenhydramine produced a significant reduction in sleep latency and night awakenings, with a marginal increase in sleep duration. Additionally, global weekly evaluations of the daytime performance favored diphenhydramine over the placebo [37].

Furthermore, Sunshine et al. evaluated the sedative effect of diphenhydramine in a group of 1295 postpartum women with sleep problems through a controlled, double-blind study. The patients were assigned to receive an oral dose of diphenhydramine hydrochloride (12.5, 25, or 50 mg), mepirizole fumarate (36, 72, or 144 mg), or a placebo. The hypnotic activity was clinically evaluated using both subjective and objective techniques. It was found that both mepirizole and diphenhydramine, at all doses, were effective hypnotics compared to the placebo, based on sleep latency, sleep duration, night-time awakenings, a global assessment, and morning alertness. Although a dose–response relationship was documented, it was also concluded that increasing the dose of these medications within the studied range produced only a minimal increase in efficacy [38].

Moreover, Kudo and Kurihara conducted a double-blind study of 144 psychiatric patients with insomnia, where diphenhydramine hydrochloride at doses of 12.5, 25, and 50 mg demonstrated clinical improvement in over 60% of patients after two weeks of treatment. Side effects were reported in 7.6% of participants but were mild, and no signs of drug dependence were observed. Treatment effectiveness was greater in patients without prior insomnia therapy, with a dose-dependent increase in hypnotic effects in this subgroup. These results suggest that diphenhydramine is effective for managing insomnia, with the optimal dose influenced by the patient’s treatment history [39]. Please refer to Table 2 for a summary of the most important clinical studies evaluating the use of diphenhydramine in the management of insomnia.

### 3.2. Pharmacokinetics of Diphenhydramine

Simons et al. investigated the pharmacokinetics of diphenhydramine in 21 subjects categorized into three groups: children, young adults, and elderly individuals. The participants were administered a diphenhydramine syrup at a dose of 1.25 mg/kg, and blood samples were collected over a 72 h period. The study revealed that the half-life (t½), area under the curve (AUC), and mean residence time (MRT) increased with age, whereas the clearance and volume of distribution decreased. Significant differences were observed in maximal plasma concentration (Cmax), but not in the time to the maximal concentration (Tmax) across age groups. The authors noted significant variations in the t½ and clearance rates between age groups [41]. These findings suggest that elderly individuals experience prolonged drug exposure, highlighting the potential need for dose adjustments to achieve an effective and safe therapeutic steady state.

Additional studies have reported that the pharmacokinetics of diphenhydramine in children (2–17 years) were studied using a weight- and age-based dosing schedule (6.25–50 mg). Cmax and AUC increased by 90% to 140% across age groups, with a tmax of 1.5 h. Oral clearance increased with age, but no maturation effect was seen after allometric scaling. Mild somnolence was the most common side effect (95%) [37]. The data are summarized in Table 3.

### 3.3. Toxic Effects of Diphenhydramine

Diphenhydramine overdose can have toxic effects, such as increased sedation and antimuscarinic effects. Clinical signs and symptoms include drowsiness, hyperpyrexia, mydriasis, fever, flushing, agitation, tremor, dystonic reactions, hallucinations, and electrocardiographic changes on the EKG, such as prolonged QRS complexes and QT intervals, as well as the appearance of a Brugada-like syndrome [40,42]. High doses, particularly in children, may result in delirium, psychosis, arrhythmias, coma, or cardiovascular collapse. Differential diagnoses for diphenhydramine intoxication include tricyclic antidepressant overdose, acetaminophen overdose, hypoglycemia, and serotonin syndrome [26,43].

Regarding drug–drug interactions, contraindicated interactions have been reported with oxybates and potassium salts [44]. The co-administration of diphenhydramine with epinephrine or norepinephrine should be avoided due to the risk of prolonged hypertension. Diphenhydramine has been reported to enhance the sedative and hypnotic effects of benzodiazepines and Z-drugs. Alcohol consumption is also discouraged while taking diphenhydramine. Concurrent use with monoamine oxidase inhibitors may intensify central nervous system depression and anticholinergic effects. Moreover, combining diphenhydramine with first- or second-generation antipsychotics (e.g., chlorpromazine, olanzapine) or antiparkinsonian agents (e.g., benztropine, trihexyphenidyl) increases the risk of additive anticholinergic effects [21,43].

Recent reports indicate a rising incidence of diphenhydramine poisoning, highlighting the need for the timely administration of physostigmine in selected cases [45,46]. Alerts have been issued regarding the “Benadryl challenge”, a social media trend linked to severe intoxications [47]. Additionally, diphenhydramine has been implicated in suicide attempts [48]. Altogether, these findings underscore the importance of not underestimating the drug’s potential for harm.

Together, there are many studies showing that diphenhydramine improves key parameters related to sleep including sleep onset latency and overall sleep quality. In addition, the rapid pharmacokinetic action of diphenhydramine allows for rapid sleep onset, which may be beneficial for patients with insomnia who need immediate intervention to promote rest. Unlike other sedative hypnotics, diphenhydramine has minimal abuse potential and a low risk of residual sedation as a side effect. These characteristics make diphenhydramine an appealing option for the short-term treatment of insomnia, particularly in adults without comorbidities, provided that the dose and duration are carefully managed to minimize potential risks. Its use should be avoided in individuals with cardiac or pulmonary conditions, or in those taking other sedatives, hypnotics, or anticholinergic agents. Use in the elderly is not recommended due to the heightened risk of severe anticholinergic effects with cumulative exposure [49].

### 3.4. Consensus Results

**Figure 1:**
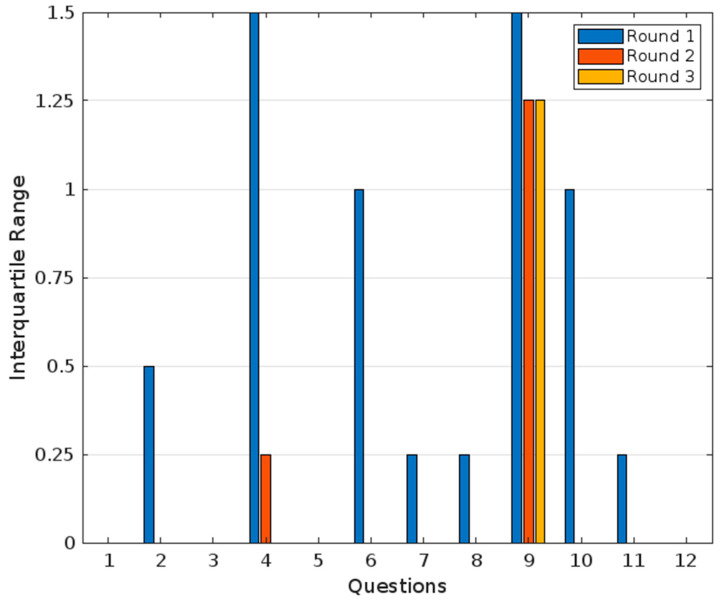
Interquartile range across rounds from expert’s panel.

The following are the results of the consensus for each dimension analyzed; results on interquartile range across the rounds are showed in Figure 1. In Chapter 1, “Evaluation of the Use of Diphenhydramine for Insomnia: Efficacy, Safety, Convenience, and Cost”, experts evaluated the following statements:

- For question 1, “Diphenhydramine is an effective medication for the management of acute insomnia”, the panel of experts unanimously agreed, giving a rating of 5/5 with an interquartile range of 0 (100% agreement). This indicates complete agreement and consensus on the premise. This unanimous consensus highlights a shared confidence in diphenhydramine’s efficacy in managing acute insomnia.
- For question 2, “Diphenhydramine is a safe medication for the management of short-term insomnia”, 80% of the experts agreed, demonstrating strong agreement with this premise. The interquartile range was 0.5, reflecting a consensus. Frequency analysis revealed that one out of five experts were neutral, four out of six partially agreed, and one out of six fully agreed. These findings suggest a consensus regarding the safety of diphenhydramine for short-term use, although the neutral stance of one expert and partial agreements indicate a need for the further exploration of specific safety concerns.
- For question 3, “If diphenhydramine were available in the Colombian market, do you consider this medication could be an accessible option for managing short-term insomnia?”, the panel showed full agreement (100%) on this statement, with a median value of five and an interquartile range of zero. These results indicate a unanimous consensus among the experts, affirming that diphenhydramine is perceived as an accessible option for managing short-term insomnia if made available in the Colombian market. This agreement reflects the experts’ confidence in its potential affordability and practicality for patients.

Grouped bars display the interquartile range across three rounds of questions. Questions 1, 3, 5, and 12 have an interquartile range of zero, indicating strong consensus with no variability among expert responses. Questions 2, 6, 7, 8, 9, and 11 show consensuses with slight variability. In contrast, questions 4 and 9 did not reach consensus in the first round due to high variability in responses. Question 4 exhibited a decrease in the interquartile range, achieving a consensus by round two. However, question 9 did not reach a consensus across all three rounds.

- For question 4, “Diphenhydramine is a convenient medication for most patients with short-term insomnia, regardless of their comorbidities or clinical situations, and therefore has the potential to be marketed as an over-the-counter medication for managing short-term insomnia”, 80% of experts agreed, showing strong agreement, but not a unanimous consensus. The median value was 4, with an interquartile range of 1.5, indicating slight variability in responses. Frequency analysis revealed that one out of six experts partially disagreed, two out of six partially agreed, and two out of six fully agreed. This variability suggests differing perspectives on the convenience of diphenhydramine, particularly regarding its suitability for patients with comorbidities or diverse clinical situations. The partial disagreement and variability highlight that while there is a general agreement, additional research or clarification may be necessary to address specific concerns.

In the second round, a consensus was achieved with an interquartile range of 0.25. Frequency analysis revealed that one of five experts remained neutral and four out five strongly agreed with the statement. The median value was 3.8, indicating that after the first round and subsequent revisions, experts agreed that diphenhydramine is a convenient medication for most patients with short-term insomnia and has the potential to be marketed as an over-the-counter medication.

In Chapter 2, “Type(s) of insomnia where diphenhydramine could be used”, the results were the following:

- For question 5, “Diphenhydramine is a useful medication for short-term insomnia (less than 3 months in duration)”, the median value was five, and the interquartile range was zero, reflecting a unanimous agreement and consensus (100% agreement). Frequency analysis revealed that all five experts rated this statement with a five, further affirming the unanimity of the consensus.
- For question 6, “Diphenhydramine is a useful medication for chronic insomnia (more than 3 months in duration)”, the median value was 1.6, and the interquartile range was 1, reflecting total disagreement and consensus (0% agreement) within the panel. Frequency analysis showed that two out of five experts rated it as one, and three out of five rated it as two. This result indicates that the panel does not recommend diphenhydramine for chronic insomnia.

In Chapter 3, “Use of diphenhydramine as a hypnotic-sedative by age group”, the results were the following:

- For question 7, “Diphenhydramine is an effective and safe medication for children aged 7 and older”, the panel showed a median value of 4.2 and an interquartile range of 0.25, reflecting unanimous agreement and consensus (100% agreement). Frequency analysis revealed that four out five experts rated it as four, and one out of five rated it as five. This indicates a consistent and strong level of agreement with the statement.
- For question 8, “Diphenhydramine is an effective and safe medication for young adults (18 to 65 years) for managing short-term insomnia”, the median value was 4.8, and the interquartile range was 0.25, reflecting a unanimous agreement and consensus (100% agreement). Frequency analysis showed that one out of five experts rated it as four, while four out of five rated it as five, demonstrating a high level of agreement with slight variability.
- For question 9, “Diphenhydramine is an effective and safe medication for elderly individuals (65 years and older) for managing short-term insomnia”, the median value was 3, and the interquartile range was 1.5, reflecting no agreement and no consensus (20% agreement). Frequency analysis indicated that two out of five experts rated it as two, two out of five as three, and one out of five as five. This wide distribution of ratings underscores the lack of consensus and varying perspectives on this statement in the first round.

Since a consensus was not achieved in the first round, a debrief was conducted on the use of diphenhydramine for elderly individuals. However, rounds two and three showed an interquartile range of 1.25, indicating significant variability in responses, and thus, no consensus was reached. In round two, frequency analysis revealed that one out of five experts partially disagreed, one out of five remained neutral, and three out of five partially agreed, with a median value of 3.4. In round three, one out of five experts partially disagreed, one out of five experts remained neutral, two out of five partially agreed, and one out of five fully agreed, with a median value of 4.2. Although there was a slight shift toward agreement, a consensus was still not achieved.

In Chapter 4, “Duration of diphenhydramine treatment for managing insomnia”, the results were the following:

- For question 10, “The maximum recommended duration for using diphenhydramine as a hypnotic/sedative for short-term insomnia should be around four weeks”, the median value was 4.6, and the interquartile range was 1, showing a strong agreement and tight consensus (100% agreement). Frequency analysis revealed that two out five experts rated it as four, while three out of five rated it as five. This indicates a shared belief in limiting the duration of diphenhydramine use, with a small degree of variability.

In Chapter 5, “Evidence and levels of evidence on the use of diphenhydramine for managing short-term insomnia”, the results were the following:

- For question 11, “There is a sufficient body of clinical evidence to recommend the use of diphenhydramine in patients with short-term insomnia”, the panel’s answers showed a median value of 4.8 and an interquartile range of 0.25, reflecting a strong agreement and consensus (100% agreement). Frequency analysis showed that one out of five experts partially agreed and four out of five strongly agreed with the statement.
- For question 12, “There is a sufficient level of clinical evidence to recommend the use of diphenhydramine in patients with short-term insomnia”, the median value was four, and the interquartile range was zero, reflecting a partial agreement and strong consensus (100% agreement). Frequency analysis revealed that all experts rated this statement as a four, emphasizing a unified agreement.

## 4. Discussion

As shown above, several studies have consistently demonstrated that diphenhydramine is effective for the treatment of short-term insomnia [25,31,32,34,38,50]. Therefore, it is important to compare its efficacy with that of other medications currently used for insomnia.

One such study, conducted by Stewart in 1987, evaluated the efficacy of diphenhydramine in comparison with temazepam. In the study, diphenhydramine was administered at a dose of 50 milligrams for five consecutive nights, with two nights of placebo between each five-day treatment period. Sleep-related metrics, including sleep quality, sleep onset latency, number of awakenings, and total sleep duration, were assessed to determine the effects of both treatments. The results indicated that diphenhydramine was as effective as temazepam as a hypnotic agent in older adults. Moreover, diphenhydramine significantly improved self-perceived sleep latency, and by the fifth night of treatment, the self-reported sleep duration was significantly longer with diphenhydramine than with temazepam. Regarding neurological adverse effects, neither diphenhydramine nor temazepam produced significant impairments [36].

In a similar study, Glass et al. compared the efficacy of temazepam and diphenhydramine. This trial was conducted over a 14-night treatment period. Both medications demonstrated hypnotic efficacy, but temazepam was more effective than diphenhydramine when compared with a placebo at the doses tested. The authors noted that this difference was offset by the increased risk of falls associated with temazepam use [35].

When comparing diphenhydramine with a non-benzodiazepine hypnotic, Katayose et al. evaluated the effects of diphenhydramine (50 milligrams), ketotifen (1 milligram), and the Z-drug zolpidem (10 milligrams). This study was a randomized, double-blind, placebo-controlled trial in which overall sleep quality, daytime sleepiness, and psychomotor performance were assessed. Among the most significant findings, diphenhydramine and zolpidem produced comparable effects on overall sleep quality. However, diphenhydramine significantly prolonged rapid eye movement (REM) sleep latency and reduced the percentage of REM sleep. Regarding daytime effects, diphenhydramine showed a tendency to increase next-day sedation and led to a significant reduction in the psychomotor performance. The authors concluded that both diphenhydramine and ketotifen significantly increased subjective and objective sleepiness while significantly impairing the next-day psychomotor performance, resulting in clinically relevant sedative/hypnotic carryover effects [51]. Some other studies have shown that diphenhydramine can impact the next-day post-administration performance [52].

Regarding safety, Erb and Bschor conducted a systematic review of the literature from 1972 to 2012 and reported a clinical case providing evidence of the addictive potential of diphenhydramine. Their findings highlight the need for caution, particularly in patients with a history of substance use disorders [53]. Other studies have also emphasized the importance of the careful use of diphenhydramine [54]. There is a similar preoccupying scenario as there is substantial evidence demonstrating the addictive potential of benzodiazepines and Z-drugs [55,56]. Overall, the long-term use of hypnotic agents carries a significant risk of dependence. While benzodiazepines and Z-drugs have been extensively studied in this regard, fewer studies have addressed the potential for diphenhydramine dependence.

Diphenhydramine is associated with several disease-related interactions. In patients diagnosed with major depressive disorder who are also taking anxiolytics, sedatives, or hypnotics, the use of diphenhydramine may lead to episodes of disinhibition, aggressiveness, agitation, or hallucinations. Individuals with comorbid conditions such as prostatic hypertrophy, urinary retention or obstruction, glaucoma, or gastrointestinal obstruction are particularly susceptible to enhanced anticholinergic effects. Moreover, diphenhydramine can increase the viscosity of bronchial secretions, potentially leading to respiratory tract obstruction; thus, caution is advised in patients with asthma or chronic obstructive pulmonary disease. Although infrequent, cardiovascular side effects have been reported, including tachycardia, palpitations, electrocardiographic abnormalities, arrhythmias, hypotension, and hypertension, particularly in individuals with pre-existing cardiac conditions [57]. Furthermore, studies have indicated that the higher cumulative use of anticholinergic medications, as diphenhydramine, is associated with an increased risk of dementia. However, to date, there is no conclusive evidence establishing diphenhydramine as a direct cause of dementia [58,59].

During pregnancy, diphenhydramine is not contraindicated, as current evidence does not suggest an increased risk of miscarriage or congenital anomalies. However, during breastfeeding, diphenhydramine may pass into breast milk in small amounts. While generally well tolerated, high doses may lead to irritability or alterations in sleep patterns in the nursing infant. Therefore, the lowest effective dose should be used and prolonged use should be avoided [60].

The expert consensus highlighted several points in favor of diphenhydramine. There was strong agreement regarding its efficacy, safety, and short duration of action, supporting its use exclusively for short-term insomnia. However, there was substantial disagreement regarding its use in chronic insomnia and in the elderly. These concerns align with available evidence, as the effectiveness of diphenhydramine has only been demonstrated for short-term insomnia. Furthermore, in older adults, all medications, including diphenhydramine, should be prescribed with caution or avoided due to potential risks. Regarding its availability as an over-the-counter medication, its approval by the Food and Drug Administration represents a significant milestone [61]. Diphenhydramine is widely accessible and affordable, making it a practical option for many individuals.

## 5. Conclusions

Diphenhydramine at a dose of 50 mg before sleep has been shown to be effective for short-term insomnia and can be used safely in healthy young and adult individuals. However, diphenhydramine should be avoided in the elderly, as well as in individuals with concurrent cardiac or pulmonary conditions, or those taking sedative/hypnotic medications. When compared to other medications, diphenhydramine demonstrates a similar efficacy profile; however, next-day side effects, such as residual sedation and cognitive impairment, are frequently reported. The available evidence and expert consensus support its use as an over-the-counter medication option for short-term insomnia. Nevertheless, patients should always be informed about its potential adverse effects, including next-day drowsiness and impaired psychomotor performance. Additionally, while the risk of dependence is lower than that of other hypnotics, its addictive potential should not be overlooked. Given these factors, diphenhydramine remains a viable short-term treatment, provided that its risks and benefits are carefully considered.

## Figures and Tables

**Table 1:** Pharmacological characteristics and clinical indications of common drugs used in insomnia.

Drug	Pharmacological Action/Group	Dose	T max	Vd	t_1/2_	Metabolism/Elimination	Indication	Use in Special Populations (Caution/Avoid)
Diphenhydramine	H1RA	12.5–50 mg	2–3 h	3.3–6.8 L/kg	2.4–9.3 h	First-pass; CYP450 isoenzymes/urine	Insomnia, allergies, nausea	Chronic liver disease, QT prolongation
Hydroxyzine	H1RA	50–100 mg	2 h	16.0 ± 3.0 L/kg	14–25 h	Liver; CYP3A4, CYP3A5/urine	Anxiety, pruritus, insomnia, allergies	Elderly, renal, and hepatic impairment
Quetiapine	D2/5-HT2A RA	25–100 mg	1.5 h	10 ± 4 L/kg	6–7 h	Liver; CYP3A4, CYP2D6/urine and feces	Psychiatric disorders, insomnia (low dose)	Young and elderly, QT prolongation
Levomepromazine	D2/H1/MRA	5–25 mg	1–2 h (est.)	16 L/kg (est.)	~20 h	Extensive first-pass; liver	Amnesia, nausea and vomiting, psychiatric disorders, insomnia (low doses)	Elderly
Temazepam	GABA-A PAM	7.5–30 mg	2–3 h	1.3–1.5 L/kg	3.5–18 h	Liver, conjugation/urine	Insomnia	Pregnancy (caution)
Triazolam	GABA-A PAM	0.125–0.5 mg	1–2 h (est.)	~1 L/kg (est.)	1.5–5.5 h	Liver, conjugation/urine	Insomnia	Elderly
Eszopiclone	GABA-A AG	1–3 mg	1 h	89.9 L	6.1 h	Liver, CYP3A, CYP2C8, CYP2E1/urine	Insomnia	Elderly
Zaleplon	GABA_B_Z	5–20 mg/day	1 h	1.4 L/kg	1 h	Aldehyde oxidase	Insomnia	Hepatic impairment
Zolpidem	GABA-A SA	5–10 mg	1.6 h	0.54–0.68 L/kg	2.5 h	Liver, CYP3A4, CYP1A2, CYP2C9/urine	Insomnia	Elderly, hepatic impairment
Amitriptyline	SERT/NETI	10–100 mg	2–12 h	1221 ± 280 L	24.65 ± 6.31 h	Liver, CYP2C19, CYP3A4, CYP2D6/urine	MDD, neuropathic pain, migraine, insomnia	Pregnancy, breastfeeding, QT prolongation
Trazodone	SERTI/5-HT2A RA	25–200 mg	8 h	0.84 ± 0.16 L/kg	7.3 ± 0.8 h	Liver, CYP3A4/urine	MDD, insomnia, anxiety	QT prolongation
Gabapentin	VGCC AI	100–600 mg	2–3 h	58 ± 6 L	5–7 h	Unchanged	Antiseizure, neuropathic pain, insomnia	Renal impairment
Melatonin	MT1/MT2 AG	1–5 mg	Variable	~1.2–1.5 L/kg (est.)	35–50 min	Liver, various	Insomnia, circadian rhythm disorders	Elderly, pregnancy (caution)
Lemborexant	OX1R/OX2RA	5–10 mg	1–3 h	1970 L	17–19 h	Liver, CYP3A4	Insomnia	Narcolepsy
Daridorexant	OX1R/OX2RA	25–50 mg	1.3 h	31 L	8 h	Liver, CYP3A4	Insomnia	Narcolepsy
Suvorexant	OX1R/OX2RA	10 mg	2 h	49 L	12 h	Liver, CYP3A4, CYP2C19	Delirium Prophylaxis, Insomnia	Narcolepsy, hepatic impairment

D2/5-HT2A RA: antagonist of the D2 dopamine receptors and the 5-HT2A serotonin receptors; D2/H1/MRA: antagonist of the D2 dopamine receptors, H1 histamine receptors, and muscarinic receptors (M); GABA-A AG: agonist of the GABA-A receptors; GABA-A PAM: positive allosteric modulator of the GABA-A receptors; GABA-A SA: selective agonist of the GABA-A receptors; H1RA: antagonist of the H1 histamine receptors; MT1/MT2 AG: agonist of the MT1 and MT2 melatonin receptors; SERT/NETI: inhibitor of the serotonin (SERT) and norepinephrine (NET) transporters; SERTI/5-HT2A RA: inhibitor of the serotonin transporter (SERT) and antagonist of the 5-HT2A serotonin receptor; OX1R/OX2RA: orexin receptor 1 and 2 antagonist. Vd: colume of distribution; VGCC AI: inhibitor of voltage-gated calcium channels. This table was generated mostly using DrugBank Open Data.

**Table 2:** Summary of the most important clinical studies evaluating the use of diphenhydramine in the management of insomnia.

Reference	Population	Design	Doses	Main Findings	Safety
Teutsch et al. (1975) [34]	More than 100 elderly patients in VA hospitals	Comparative with placebo	50 mg and 150 mg	It was not significantly different from pentobarbital for control of insomnia	No significant differences in adverse effects
Russo et al. (1976) [37]	50 children with sleep disorders	Placebo controlled	10 mg/kg	Significantly reduced sleep latency and night awakenings	Significantly reduced sleep latency and night awakenings
Carruthers et al. (1978) [27]	6 healthy volunteers	Double blind, crossover	50 mg	Positive correlation between plasma concentration and sedative effects	No specific adverse effects are detailed
Sunshine et al. (1978) [38]	1295 postpartum women with insomnia	Double-blind controlled study	12.5, 25, and 50 mg	Effective hypnotics in comparison to placebo	No significant adverse events reported
Rickels et al. (1983) [31]	111 patients with mild to moderate insomnia	Double blind, crossover	50 mg	Improved several sleep parameters, patients reported feeling more rested	More side effects reported with diphenhydramine
Stewart et al. (1987) [36]	17 nursing home residents with insomnia	Double blind, crossover	50 mg	Shorter sleep latency and longer sleep duration than temazepam	Worse performance on neurological tests compared to placebo
Roth et al. (1987) [28]	16 healthy adults	Crossover	50 mg (3 times a day)	No significant difference compared to loratadine	Daytime sedation and decreased performance
Borbély et al. (1988) [25]	10 young and healthy adults	Double blind, crossover	50 mg and 75 mg	No significant differences in subjective sleep parameters compared to placebo	Diphenhydramine did not cause deterioration in psychomotor performance or rebound insomnia
Kudo and Kurihara (1990) [39]	144 psychiatric patients aged 15 to 82 years old with insomnia	Randomized, Double blind	12.5, 25, and 50 mg	Diphenhydramine was effective in improving sleep quality in psychiatric patients	Well tolerated, no serious side effects during the trial
Roehrs et al. (1993) [30]	12 young and healthy men	Double blind, Latin square	50 mg	Significant sedative effects for 6.5 h, similar to triazolam	Residual sedation for ethanol but not for diphenhydramine and triazolam
Schweitzer et al. (1994) [33]	12 atopic subjects	Double blind, crossover	50 mg (3 times a day)	Decreased alertness and performance on day 1, tolerance developed by day 3	Central nervous system depression only on the first day
Richardson et al. (2002) [40]	15 healthy volunteers aged 18–50 years	Randomized, double-blind, crossover	50 mg (2 times a day)	Increased drowsiness on day 1, tolerance developed by day 4	Performance decline reversed on day 4
Morin et al. (2005) [32]	184 patients with mild insomnia	Multicenter, randomized, placebo-controlled	50 mg (2 times a day)	Improvements in subjective sleep parameters, increased sleep efficiency in the first 14 days	There were no significant residual effects or serious adverse events.
Glass et al. (2008) [35]	25 elderly with insomnia	Latin Square Desing	50 mg	Improvement only in the number of awakenings compared to placebo; no better than temazepam	Similar number of adverse events, one fall reported with temazepam
Moulin et al. (2022) [29]	27 healthy adult participants	Randomized, double-blind, placebo-controlled, crossover	50 mg for 7 days	Improvement in sleep debt, natural supplement showed better efficacy in sleep parameters	No serious adverse effects

**Table 3:** Diphenhydramine pharmacokinetic parameters by age group.

Parameter	Children (8.9 ± 1.7 y.o.)	Young Adults (31.5 ± 10.4 y.o.)	Elderly (69.4 ± 4.3 y.o.)
Weight (kg)	31.6 ± 6.8	70.3 ± 9.9	71.0 ± 11.4
Dose (mg)	39.5 ± 8.4	87.9 ± 12.4	86.0 ± 7.3
Cmax (ng/mL)	81.8 ± 30.2	133.2 ± 37.6	188.4 ± 54.5
Tmax (h)	1.3 ± 0.5	1.7 ± 1.0	1.7 ± 0.8
t½ (h)	5.4 ± 1.8	9.2 ± 2.5	13.5 ± 4.2
Cl (mL/min/kg)	49.2 ± 22.8	23.3 ± 9.4	11.7 ± 3.1
Vdss (L/kg)	17.9 ± 5.9	14.6 ± 4.0	10.2 ± 3.0
Vd (L/kg)	21.7 ± 6.6	17.4 ± 4.8	13.6 ± 6.3
AUC (ng/mL/h)	475 ± 137	1031 ± 437	1902 ± 572
MRT (h)	6.4 ± 1.6	11.3 ± 3.1	14.8 ± 2.8

Cmax: Maximum plasma concentration; Tmax: Time to reach maximum plasma concentration; t½: Elimination half-life; Cl: Clearance (mL/min/kg); Vdss: Volume of distribution at steady state (L/kg); Vd: Volume of distribution (L/kg); AUC: Area under the concentration–time curve (ng/mL/h). Table adapted from [41].
